# Synergistic Enhancement of Straw Hydrolysis and Lactic Acid Production in *Talaromyces pinophilus* Through Combined Random Mutagenesis and Plasmid Reconstruction

**DOI:** 10.3390/jof12060405

**Published:** 2026-06-03

**Authors:** Siyuan Yue, Ya Li, Peng Li, Jing Zeng, Junhui Nie, Cheng Zhang, Tong Wang, Jianjun Guo, Lin Yuan

**Affiliations:** Institute of Biomanufacturing, Jiangxi Academy of Sciences, Nanchang 330095, China; jxskxyysy@126.com (S.Y.); liya0104@126.com (Y.L.); pengli_jx@126.com (P.L.); zengjingwhu@126.com (J.Z.); niejunhui_jxas@163.com (J.N.); zhang_chengs@163.com (C.Z.); m17770660365@163.com (T.W.); guojianjunjxas@163.com (J.G.)

**Keywords:** *Talaromyces pinophilus*, cellulase, lignocellulose biomass, consolidated bioprocessing

## Abstract

Lignocellulosic biorefineries are limited by the high cost of cellulolytic enzymes. Consolidated bioprocessing (CBP), which integrates saccharification and fermentation in one step, offers a solution to this challenge. In this study, a cellulase-hyperproducing mutant of *Talaromyces pinophilus*, Y117, was generated from the parental strain TP117 via sequential ultraviolet irradiation and NTG (N-methyl-N′-nitro-N-nitrosoguanidine) mutagenesis. Enzymatic secretion and lignocellulose degradation capacities were evaluated, focusing on agricultural residues, particularly corncob. Y117’s performance was compared with TP117 and *Trichoderma reesei* Rut-C30 (TR30) under high-solids fermentation. Furthermore, the lactate dehydrogenase A (*ldhA*) gene from *Rhizopus oryzae* was heterologously expressed in Y117 to direct hydrolyzed sugars toward lactic acid (LA). Y117 exhibited significantly enhanced enzymatic secretion, achieving FPase activity of 8.9 IU/mL and a substrate utilization rate of 72.2% at 125 g/L corncob solids. Y117 outperformed TP117 and TR30 in cellulase, xylanase, and CMCase activities, as well as growth under high-solids fermentation conditions. In the LA fermentation process, Y117 produced 14.20 g/L LA, a notable improvement compared to TP117 (5.33 g/L) and TR30 (2.71 g/L). While LA productivity and yield currently remain below bacterial benchmarks, the unique CBP capability of Y117 provides a foundation for further metabolic engineering toward industrial viability. The engineered *T. pinophilus* Y117 demonstrates promising potential as a CBP platform for efficient straw-to-LA conversion, providing a sustainable approach for third-generation biobased materials production.

## 1. Introduction

Biobased materials have emerged as a cornerstone of the modern green economy, with wide applications in packaging, textiles, biomedical engineering, and other fields [1]. Among them, lactic acid (LA) serves as the primary monomer for polylactic acid (PLA), a biodegradable polymer known for its excellent mechanical strength, thermal stability, and biocompatibility [2,3]. LA is typically produced through bacterial homofermentation or fungal heterofermentation of glucose [4,5], which offers high conversion efficiency but relies heavily on starch-derived sugars as feedstocks [6]. This reliance on food-based resources not only undermines cost competitiveness with petroleum-derived plastics but also raises concerns over competition with human food supplies [7]. As global demand for sustainable and eco-friendly materials grows, it is crucial to develop non-food-based methods for LA biosynthesis.

Several strategies have been explored for non-food-based LA production, including the use of glycerol [8], a byproduct of biodiesel refining, or sugars derived from agricultural residues like straw [9]. Due to their abundance, renewability, and low cost, straw-derived sugars have received significant attention. Microbial engineering efforts focus on enhancing the ability of strains to utilize mixed saccharides for efficient LA fermentation [10]. For instance, engineered *Aspergillus brasiliensis* expressing *ldhA* showed enhanced LA production from mixed sugars [11], and gene-edited strains of *Saccharomyces cerevisiae* and *Escherichia coli* also exhibited improved fermentation on hydrolysates [12,13]. Despite such advances, the straw-to-sugar route remains technically and economically challenging due to multistep pretreatment and hydrolysis, including costly cellulase fermentation, harsh chemical pretreatment, enzymatic saccharification, and sugar concentration [14,15]. These steps not only drive-up costs but also pose environmental risks. These challenges underscore the need for consolidated bioprocessing (CBP) approaches that bypass enzyme production and pretreatment [16,17].

CBP provides an attractive strategy by integrating saccharification and fermentation into a single step, directly converting the cellulose and hemicellulose fractions of straw into LA without the need for external enzymes [18]. Significant progress has been made toward realizing this approach. For example, researchers at Dalian University of Technology constructed a cattle stomach–derived microbial consortium capable of one-pot conversion of H_2_SO_4_-pretreated corn stover into LA, producing 43.73 g/L LA with a yield of 0.50 g/g under constant pH control at 5.5 [19]. Similarly, BluCon Biotech GmbH reported direct cellulose-to-LA conversion by a novel thermophilic *Caldicellulosiruptor* strain, producing 70 g/L L-LA with 99.4% purity and a productivity of 1.0 g/L/h from 200 g/L microcrystalline cellulose at 70 °C, maintaining pH at 6.4 [20]. Despite these advances, the need for stringent pH regulation and substrate pretreatment highlights the technical and economic barriers that hinder cost-effective lignocellulose utilization. Furthermore, CBP systems based on mixed microbial consortia often experience prolonged fermentation cycles and unstable community structures, limiting their industrial scalability. Therefore, developing controllable, single-strain CBP platforms with shorter fermentation cycles and enhanced acid resistance is essential for advancing next-generation industrial LA production.

*Talaromyces pinophilus* is a filamentous ascomycete recognized for its ability to secrete diverse lignocellulolytic enzymes, including cellulases, hemicellulases, and β-glucosidases [21]. Notably, the β-glucosidase of *T. pinophilus* exhibits remarkable acid tolerance with a pH optimum around 4.0, a highly desirable trait for consolidated bioprocessing under acidic conditions [22]. In addition to its enzymatic capacity, *T. pinophilus* can naturally produce organic acids such as lactic acid during lignocellulose utilization, indicating inherent metabolic pathways relevant to biobased chemical production [23]. These characteristics establish *T. pinophilus* as a promising platform for both enzyme production and consolidated bioprocessing of lignocellulosic biomass.

In a previous work, *Talaromyces pinophilus* TP117 was isolated from paddy soil samples in Jiangxi Province [24]. This study aimed to generate a cellulase-hyperproducing mutant via sequential UV and NTG mutagenesis. The resulting strain, Y117, displayed strong intrinsic straw-degrading ability. We further aimed to evaluate its cellulase secretion and degradation efficiency on the three major lignocellulosic resources—rice straw, corncob, and wheat straw—under high-solids conditions. Finally, we aimed to redirect carbon flux from straw-derived sugars toward lactic acid biosynthesis by heterologously expressing *ldhA* from *Rhizopus oryzae*. This approach creates a high-performance fungal chassis for direct straw-to-LA conversion and provides a new microbial route for third-generation biobased material production.

## 2. Materials and Methods

### 2.1. Fungal Strain and Mutagenesis

The parental strain *T. pinophilus* TP117 was isolated from soil in Jiangxi province, China. It was inoculated on Potato Dextrose Agar (PDA, P8931, Beijing Solarbio Science & Technology Co., Ltd., Beijing, China) plates at 30 °C. Mycelia were scraped into sterile water, and the concentration was adjusted to 10^8^ CFU/mL by blood cell counting plate method. A 5 mL mycelial suspension was inoculated onto a sterile PDA plate, then UV-irradiated for 30 s at a distance of 10 cm, resulting in a 90% drop in viability. The irradiated suspension was spread onto PDA plates containing 0.1% AZCL-HE-cellulose (Megazyme, Wicklow, Ireland), and incubated at 30 °C. Colonies with cellulolytic activity were identified by the blue halo formed around them, the colony circle and color-changing circle diameter were measured, and the colony circle to color-changing circle diameter (CCD) ratio was regarded as an effective indicator of cellulase secretion capacity. Each positive colony was cultured in shake flasks, and enzyme activity was assessed as described below. The strain having the highest FPase activity was selected for NTG mutagenesis. The diluted solution containing the mycelium was treated with 0.05% (*w*/*v*) NTG for 30 min, with a resultant 90% drop in viability. After exposure, mycelia were collected by centrifugation (8000 rpm, 4 °C) and washed twice with sterile water. The mycelia were then spread onto PDA plates containing AZCLHE-cellulose for screening as described [24]. *Trichoderma reesei* Rut-C30 (TR30) was obtained from the China General Microbiological Culture Collection Center.

### 2.2. Cultivation and Medium

The composition of the media used for shake flask and fermenter cultures is as follows: 40 g/L (preculture) or 50 g/L (production culture) microcrystalline cellulose (CAS 9004-34-6; BBI Life Sciences, Shanghai, China) as a cellulose source; 24 g/L KH_2_PO_4_; 1 g/L Tween 80; 5 g/L (NH_4_)_2_SO_4_; 1.2 g/L MgSO_4_·7H_2_O; 0.01 g/L ZnSO_4_·7H_2_O; 0.01 g/L MnSO_4_·6H_2_O; 0.01 g/L CuSO_4_·7H_2_O; 2 g/L (preculture) or 4 g/L (production culture) urea. The pH was adjusted to 4.0 with H_2_SO_4_ and KOH before sterilization. This pH was selected as optimal for cellulase induction and activity in *T. pinophilus* [24]. Distilled water and all components, except for trace elements (ZnSO_4_·7H_2_O, MnSO_4_·6H_2_O, CuSO_4_·7H_2_O) and urea, were sterilized at 121 °C for 20 min. For fermentation, corncob, rice straw or wheat straw (at concentrations of 50 g/L, 75 g/L, 100 g/L, 125 g/L, and 150 g/L) were used as carbon sources instead of microcrystalline cellulose. Corncob, rice straw and wheat straw materials were ground and passed through a 100-mesh sieve.

### 2.3. Batch Fermentation

Colonies of Y117, TP117 and TR30 were each inoculated into 250 mL Erlenmeyer flasks containing 50 mL of preculture medium and incubated at 30 °C with shaking (235 rpm) for 5 days to activate the seeds (mycelial precultures). The activated seed cultures were then transferred to 1 L Erlenmeyer flasks containing 200 mL of culture medium, with corncob, rice straw or wheat straw as the carbon source. The fermentation cultures were incubated at 30 °C with shaking (235 rpm) for 7 days. Enzyme activity and fungal growth were measured by sampling every 24 h.

### 2.4. Enzymatic Assays

Filter-paper (FPase) activity was measured according to the standard IUPAC method recommended by Ghose [25]. A 50 mg filter paper strip was incubated with 0.5 mL of appropriately diluted enzyme and 1.0 mL citrate buffer (pH 4.8) in a total volume of 1.5 mL at 50 °C for 60 min. The dilution was chosen to keep glucose release within the linear range. The reaction was terminated with 3 mL DNS reagent, boiled for 5 min, and absorbance measured at 540 nm. One IU was defined as the amount of enzyme releasing 1 μmol glucose equivalent per minute. Glucose standards (0.25–2.0 mg) were run in parallel with each assay. Activity was calculated only from dilutions yielding glucose release within the 2.0 mg range and multiplied by the dilution factor.

Carboxymethylcellulase (CMCase) activity was determined using the method of Mandels et al. [26]. Briefly, 0.5 mL of carboxymethylcellulose (CMC, 2% *w*/*v*) in citrate buffer (50 mM, pH 4.8) was mixed with an equal volume of appropriately diluted enzyme solution, and incubated at 50 °C for 30 min. One IU was defined as the amount of enzyme releasing 1 μmol glucose equivalent per minute. The reducing sugars released were quantified by the dinitrosalicylic acid (DNS) assay [27].

Xylanase activity was assayed in a 1.0 mL reaction mixture containing 1% (*w*/*v*) birchwood xylan (Sigma-Aldrich, St. Louis, MO, USA) and 50 mM acetate buffer (pH 5.5), with appropriately diluted enzyme solution [28]. One IU was defined as the amount of enzyme releasing 1 μmol xylose equivalent per minute.

### 2.5. Analysis of Fermentation Products, Biomass Composition, and Extracellular Protein

The organic acids (formic acid, acetic acid, propionic acid, citric acid, and lactic acid) and ethanol present in the fermentation broth were analyzed using a Waters e2695 Alliance high-performance liquid chromatography (HPLC) system equipped with a Waters 2414 differential refractive index detector (Waters Corporation, Milford, MA, USA). Chromatographic conditions: TOSOH OApak-A column (300 mm × 7.8 mm, 7 μm) equipped with Tskgel OApak-P precolumn; column temperature: 30 °C; injection volume: 10 μL; flow rate: 1.0 mL/min; mobile phase: 0.75 mmol/L H_2_SO_4_. Calibration curves (R^2^ > 0.999) were constructed for each analyte. Detection limits were 0.01 g/L for organic acids and 0.02 g/L for ethanol.

The composition of corncob, rice straw, and wheat straw was determined using the Van Soest method with an ANKOM220 Fiber Analyzer (ANKOM Technology, New York, NY, USA). All measurements of cellulose, hemicellulose, and lignin contents were conducted in triplicate independent experiments, and the results are reported as mean values.

The total extracellular secreted protein concentration in the fermentation supernatant was determined using the bicinchoninic acid (BCA) protein assay kit (Beyotime Biotechnology, Shanghai, China) according to the manufacturer’s instructions. Briefly, fermentation broth was centrifuged at 10,000 rpm for 10 min at 4 °C, and the supernatant was collected. A 20 μL aliquot of appropriately diluted supernatant was mixed with 200 μL of BCA working reagent and incubated at 37 °C for 30 min. Absorbance was measured at 562 nm using a microplate reader. Bovine serum albumin (BSA) was used as the standard protein to construct a calibration curve (0–0.5 mg/mL, R^2^ > 0.999). All measurements were performed in triplicate.

### 2.6. MTT Cell Viability Assay

The MTT (3-(4,5-dimethylthiazol-2-yl)-2,5-diphenyltetrazolium bromide) assay has been previously validated for assessing metabolic activity and viability in filamentous fungi, including *Aspergillus* and *Trichoderma* species [29,30]. While this method measures metabolic activity (NAD(P)H-dependent reduction of MTT to formazan) rather than direct biomass, it provides a rapid and reproducible surrogate for growth assessment under high-solids fermentation conditions where gravimetric methods are technically challenging. An MTT solution (5 mg/mL) was prepared by dissolving 0.5 g MTT in 100 mL phosphate-buffered saline (PBS), then filtered through a 0.22 μm sterile membrane. The solution was stored in the dark at 4 °C. For the assay, 1 mL of fermentation solution was centrifuged at 5000 rpm for 5 min, washed with PBS, and resuspended. Cells were then incubated with 10% (*v*/*v*) MTT solution at 30 °C for 4 h. Formazan crystals were dissolved by sequential treatments: centrifugation to remove the supernatant, washing with DMSO, and final dissolution in 400 μL DMSO by incubating at 50 °C for 1.5 h. Absorbance was quantified at 490 nm using a microplate reader.

### 2.7. Heterologous Gene Expression Plasmid Construction

To construct the plasmid pBIP-TPcbh1-LDHA-cbh1T: The pBIP vector, containing an *E. coli* replication origin and an ampicillin resistance marker, was double-digested with restriction enzymes *Hind*III and *Sac*I to generate a linearized vector. The expression cassette TPcbh1-LDHA-cbh2T was obtained by overlap extension PCR (SOE-PCR). The purified PCR product was then assembled with the *Hind*III/*Sac*I-digested pBIP vector using seamless in vitro cloning (Takara, In-Fusion HD Cloning kits) to generate the intermediate plasmid pBIP-TPcbh1-LDHA-cbh1T. The intermediate plasmid was then double-digested with *Sac*I and *Bam*HI to generate a linearized vector. The TPpk-hyg-cbh1T expression cassette was similarly obtained by SOE-PCR. The purified PCR product was then assembled with the *Sac*I/*Bam*HI-digested pBIP-TPcbh1-LDHA-cbh1T vector to generate the heterologous expression plasmid pBIP-TPcbh1-LDHA-TPpk-hyg. The construction of pBIP-TR30cbh1-LDHA-TR30pk-hyg followed a similar process, with the *ldhA* gene codon-optimized for *T. pinophilus* and *T. reesei*, respectively.

### 2.8. Preparation and Transformation of Protoplasts

Colonies of Y117, TP117 and TR30 were inoculated into 250 mL Erlenmeyer flasks containing 50 mL YPD (1% yeast extract, 2% peptone, and 2% dextrose) medium and incubated at 30 °C with shaking (180 rpm) for 4 days. The mycelium was collected by centrifugation at 4000 rpm for 5 min. After washing twice with sterile water, the mycelium was suspended into 20 mL enzymatic hydrolysis buffer (0.8 M NaCl, 10 mM KH_2_PO_4_, pH 6.0) containing 0.04 g Yatalase Plus enzyme (Code No. T030, Takara Bio Inc., Shiga, Japan). The suspension was incubated at 30 °C overnight. Protoplasts were collected by centrifugation (4000 rpm, 10 min) and washed twice in Solution A (1.2 M sorbitol, 10 mM Tris-HCl, pH 7.5, 50 mM CaCl_2_).

For transformation, 10 μg of plasmid DNA was mixed with 100 μL of protoplast suspension (1 × 10^8^ protoplasts/mL) and incubated on ice for 5 min. Then, 100 μL of Solution B (40% PEG4000, 10 mM Tris-HCl, pH 7.5, 50 mM CaCl_2_) was added and the mixture incubated on ice for 30 min. Subsequently, 8.5 mL of Solution A was added and the mixture was centrifuged (4000 rpm, 10 min) to collect the protoplasts. The protoplast-plasmid mixture (50 μL) was spread onto YPSA plates (1% yeast extract, 1% tryptone, 1M sucrose, 1.2% agar) containing 500 μg/mL hygromycin and incubated upside down in the dark at 30 °C for 2–3 nights [31].

Western blot analysis was performed to detect heterologous LDHA protein expression. Total intracellular protein was extracted from mycelia using RIPA buffer, separated by 12% SDS-PAGE, and transferred to PVDF membranes. The membrane was blocked with 5% skim milk and probed with anti-His tag antibody (1:2000 dilution, Sigma-Aldrich). A horseradish peroxidase-conjugated secondary antibody was used for detection.

### 2.9. Statistical Analysis

All experiments were performed with three independent biological replicates, unless otherwise specified. Results are presented as mean ± standard deviation (SD). The IBM SPSS Statistics (version 24.0) was used for data significance analysis.

## 3. Results and Discussion

### 3.1. Isolation of Strain Y117

Following UV mutagenesis, 25 colonies exhibiting hydrolysis halos were selected and further screened on PDA plates supplemented with 0.1% AZCL-HE-cellulose to assess cellulolytic activity. Five mutants displayed significantly enhanced cellulase production. Among these, the YP003 mutant showed higher FPase activity than both the parent strain TP117 and the other mutants. To further improve cellulase yield, YP003 was subjected to chemical mutagenesis using NTG. From 15 NTG-induced positive colonies, five strains exhibited a further increase in FPase activity. These strains were cultured in shake flasks, and their cellulase production was evaluated in triplicate. Under corncob substrate cultivation, mutant Y117 achieved peak enzymatic performance, with FPase activity of 8.9 ± 0.7 IU/mL, representing a 6-fold enhancement over the parental strain TP117 after 6 days of incubation at 30 °C.

**Figure 1:**
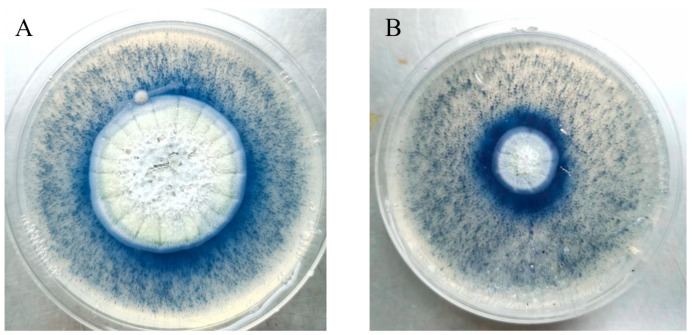
Plate assay on PDA with 0.1% AZCL-HE-cellulose after 4 days of incubation at 30 °C. The blue transparent zone indicates cellulolytic activity. (**A**) Parental strain *Talaromyces pinophilus* TP117; (**B**) Mutant strain Y117.

Colony morphology differed markedly between Y117 and TP117. TP117 formed large (4.5–4.7 cm), folded white colonies, while Y117 produced smaller (2.7–2.9 cm), hairy colonies with reddish-brown edges. The colony circle to color-changing circle diameter (CCD) ratio served as an effective indicator of cellulase secretion capacity, with lower ratios correlating with higher enzymatic activity (Figure 1). Y117 exhibited a significantly lower CCD ratio (0.6551) compared to TP117 (0.8936), which aligned with its superior FPase activity. Microscopic analysis confirmed that Y117 had identical mycelial structures to TP117, verifying it as a stable mutant. The complete genome of *T. pinophilus* Y117 has been deposited in NCBI, providing a genomic basis for its high cellulase production.

Through sequential random mutagenesis (UV irradiation followed by NTG treatment), we developed *Talaromyces pinophilus* Y117, which demonstrated significantly enhanced FPase activity compared to the parental strain TP117 in both shake-flask and batch cultures. Consistent with our previous study [24], strain Y117 exhibits significantly enhanced activities of multiple extracellular enzymes, including FPase and β-glucosidase, compared to reference strains. Genomic analysis further revealed a markedly expanded CAZyme repertoire in Y117 (2026 genes), including a substantial increase in glycoside hydrolases and carbohydrate-binding modules, supporting its enhanced capacity for extracellular enzyme production and lignocellulose degradation.

Compared with a previously reported mutant strain (*T. pinophilus* EMM), which achieved 7.3 IU/mL FPase after 10 days of fed-batch fermentation [21], Y117 attained a higher activity (8.9 ± 0.7 IU/mL) within only 6 days under batch conditions. This improved productivity, together with successful mutagenesis-based enhancements reported in other cellulolytic fungi such as *Trichoderma harzianum* and *Aspergillus uvarum* [32,33], highlights both the industrial potential of Y117 and the continued effectiveness of random mutagenesis as a practical strategy for strain improvement. Collectively, these results demonstrate that classical mutagenesis remains a robust approach for developing fungal strains with enhanced cellulase production.

### 3.2. The Effect of Different Substrate Concentrations on the Utilization of Lignocellulose by Strain Y117

**Figure 2:**
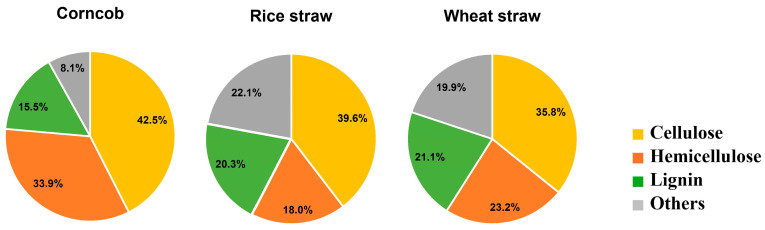
The composition of corncob, rice straw and wheat straw.

Corncob, rice straw, and wheat straw are key lignocellulosic feedstocks widely used for biofuel and biochemical production. Compositional analysis (Figure 2) revealed distinct differences in their structural carbohydrate and lignin contents. Corncob demonstrated the most favorable composition for bioconversion, containing the highest percentages of cellulose (42.5%) and hemicellulose (33.9%), along with the lowest lignin content (15.5%). In comparison, rice straw had higher cellulose content than wheat straw (39.6% vs. 35.8%), but lower hemicellulose levels (18.0% vs. 23.2%). Both straw varieties exhibited comparable lignin contents, with rice straw at 20.3% and wheat straw at 21.1%.

**Figure 3:**
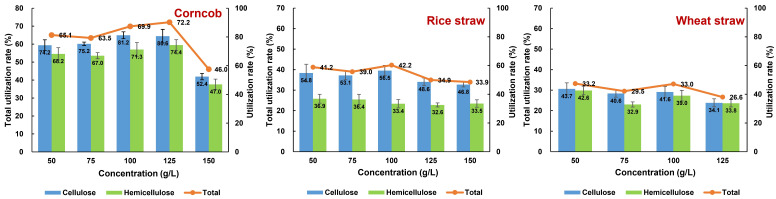
Utilization of varying concentrations of corncob, rice straw, and wheat straw by strain Y117. Blue column: cellulose utilization rate (%); green column: hemicellulose utilization rate (%); orange line: total utilization rate (%). Cellulose and hemicellulose utilization rate were calculated as the decrease determined by the Van Soest method. Data are presented as mean ± SD (*n* = 3).

The ability of strain Y117 to utilize lignocellulosic biomass was evaluated using corncob, rice straw and wheat straw as substrates at concentrations ranging from 50 g/L to 150 g/L. As shown in Figure 3, strain Y117 efficiently utilized corncob compared to rice straw and wheat straw. The total utilization rate (the percentage of initial dry substrate weight lost after fermentation) for corncob exceeded 60% at concentrations from 50 to 125 g/L, peaking at 72.2% at 125 g/L. However, at 150 g/L, the total utilization rate dropped to 46.0%, likely due to the high substrate concentration reducing fluidity and thereby limiting enzymatic hydrolysis efficiency. Strain Y117 exhibited similar total utilization rates for rice straw and wheat straw, with rice straw being slightly more efficiently utilized due to its higher cellulose content. At concentrations between 50 and 100 g/L, the total utilization rates remained stable at 39.0–42.2% for rice straw and 29.5–33.2% for wheat straw. As shown in Figure 2, cellulose and hemicellulose account for a major proportion of lignocellulosic biomass, particularly in corncob (76.4%). The high utilization of these components by strain Y117 contributes to its overall efficient degradation of corncob.

### 3.3. Comparison of Utilization Rates of Corncob, Rice Straw and Wheat Straw by Y117, TP117, TR30

As described in Section 3.2, strain Y117 demonstrated optimal utilization of corncob (125 g/L), rice straw (100 g/L), and wheat straw (100 g/L). To further investigate its performance, batch fermentation experiments were conducted to compare the lignocellulose utilization and cellulase production capabilities of Y117 with that of the parental strain *T. pinophilus* (TP117) and *T. reesei* Rut-C30 (TR30) using these three substrates as carbon sources.

**Figure 4:**
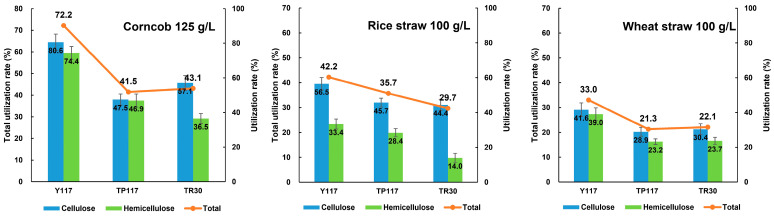
Utilization rates of Y117, TP117 and TR30 in corncob (125 g/L), rice straw (100 g/L), and wheat straw (100 g/L). Blue column: cellulose utilization rate (%); green column: hemicellulose utilization rate (%); orange line: total utilization rate (%). Cellulose and hemicellulose utilization rate were calculated as the decrease determined by the Van Soest method. Data are presented as mean ± SD (*n* = 3).

As shown in Figure 4, strain Y117 exhibited significantly higher lignocellulose utilization efficiency than TP117 and TR30 across all three biomass substrates. Specifically, in corncob cultures, Y117 achieved a cellulose utilization rate of 80.6% and hemicellulose utilization of 74.4%, outperforming TP117 (47.5% cellulose, 46.9% hemicellulose) and TR30 (43.1% cellulose, 36.5% hemicellulose). This trend was consistent for both rice straw and wheat straw cultures, where Y117 again showed the highest cellulose and hemicellulose utilization rates. Both TP117 and TR30 exhibited comparable enzymatic hydrolysis performance on rice straw and wheat straw.

**Figure 5:**
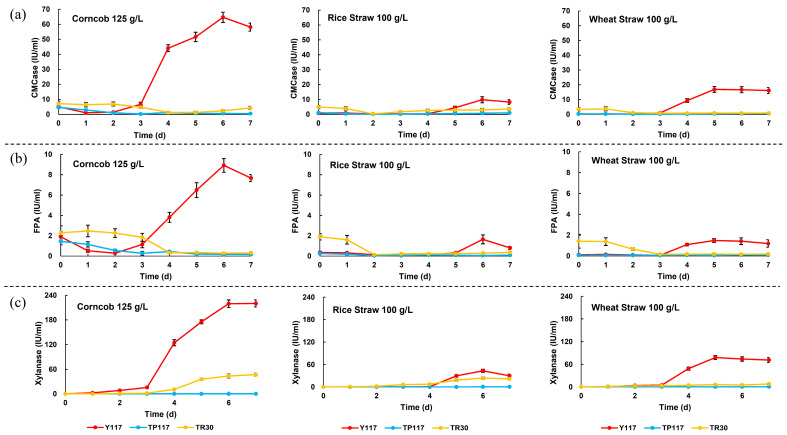
Time course of enzyme production by Y117, TP117 and TR30 in cultures of corncob (125 g/L), rice straw (100 g/L), and wheat straw (100 g/L). (**a**) CMCase; (**b**): FPase; (**c**): xylanase. Data are presented as mean ± SD (*n* = 3).

The enzyme production profiles of strains Y117, TP117, and TR30 during batch fermentation are compared in Figure 5. On corncob, Y117 exhibited a rapid increase in CMCase, FPase, and xylanase activities from day 3, reaching peak values of 64.7 IU/mL, 8.9 IU/mL, and 219.3 IU/mL, respectively, by day 7. In contrast, wheat straw cultures showed delayed enzyme production initiation (day 4), with lower peak values (CMCase: 16.9 IU/mL; FPase: 1.5 IU/mL; xylanase: 78.2 IU/mL). Rice straw cultures had an even further delay in enzyme production (day 4) and significantly lower peak activities (CMCase: 9.8 IU/mL; FPase: 1.6 IU/mL; xylanase: 43.2 IU/mL) compared to corncob cultures. On the other hand, the parental strain TP117 produced substantially lower enzyme activities across all three substrates, with particularly negligible xylanase activity.

**Figure 6:**
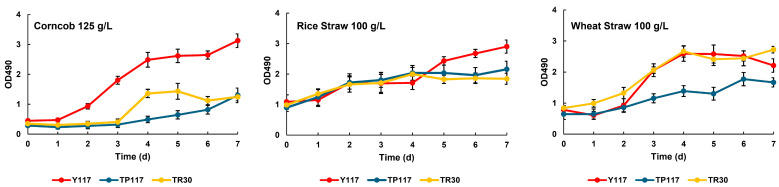
Determination of cell growth (OD490) of Y117, TP117, and TR30 in cultures of corncob, rice straw, and wheat straw. Data are presented as mean ± SD (*n* = 3).

Y117 also displayed superior growth kinetics (Figure 6), reaching a maximum OD490 of 3.13 within 7 days, representing 141% and 151% increase compared to TP117 (1.30) and TR30 (1.25), respectively. This growth performance further supports Y117’s exceptional adaptation to high-solids fermentation conditions.

In addition to enzyme activities, we evaluated the total extracellular secreted protein and specific FPase activity of the parental strain TP117 and the mutant Y117 using the BCA assay. The total extracellular protein concentration of TP117 was 0.8 mg/mL, while that of Y117 reached 8.9 mg/mL, representing an 11-fold increase. The specific FPase activity (per mg secreted soluble protein) was 0.55 IU/mg for TP117 and 1.0 IU/mg for Y117, corresponding to a 1.8-fold improvement. These results demonstrate that Y117 not only secretes substantially larger quantities of total protein but also produces cellulases with higher specific activity, further supporting its exceptional secretory capacity and industrial potential.

The compositional analysis revealed corncob as the most favorable substrate, exhibiting a superior biochemical profile with 42.5% cellulose and 33.9% hemicellulose content, along with significantly reduced lignin content (15.5%) compared to rice straw and wheat straw (Figure 2). These findings corroborate previous studies on lignocellulosic biomass composition [34,35]. Notably, our engineered strain Y117 demonstrated exceptional lignocellulose utilization capabilities, outperforming both the parental strain TP117 and the industrial benchmark *Trichoderma reesei* Rut-C30 (TR30) across all tested substrates. Specifically, strain Y117 achieved remarkable corncob utilization rate exceeding 60% within the 50–125 g/L concentration range, peaking at 72.2% at the highest substrate loading (125 g/L) (Figure 3). For rice straw and wheat straw, at their optimal concentration (100 g/L), Y117 maintained substantial utilization rates of 42.2% and 33.0%, respectively (Figure 4). Of particular significance, Y117’s performance surpassed even genetically enhanced reference strains. For instance, the Xyr1-overexpressing *T. reesei* QE2X strain, as demonstrated by Shen et al. (2022), achieved only 37.87% utilization of acid-pretreated corncob residue at 50 g/L—markedly lower than Y117’s utilization rate at 125 g/L corncob (no pretreatment) [36]. This exceptional performance under high-solids conditions underscores Y117’s outstanding potential for industrial lignocellulosic biorefining applications.

The superior lignocellulose utilization rate of Y117 stems from its dual capacity for enhanced enzymatic secretion and robust growth kinetics under high-solids conditions. As shown in Figure 5, Y117 showed substantially elevated extracellular enzyme activities compared with both TP117 and TR30 across all substrates: corncob (125 g/L), rice straw (100 g/L), and wheat straw (100 g/L). This coordinated enzymatic synergy, particularly the 21.5-fold higher CMCase activity (64.7 IU/mL vs. TP117’s 2.9 IU/mL at 125 g/L corncob), effectively overcomes lignocellulosic recalcitrance through simultaneous cellulose (FPase) and hemicellulose-lignin matrix (xylanase) deconstruction [37]. The enzymatic profile not only facilitates substrate deconstruction but also mitigates product feedback inhibition [38]. In addition to this enzymatic advantage, Y117 showed remarkably improved growth kinetics, with a substantially higher OD490 than the parental and reference strains (Figure 6). This accelerated growth reflects Y117’s enhanced capacity for both substrate assimilation and tolerance to inhibitors (e.g., furfural, organic acids), characteristic of high-solids fermentation environments [39]. While reference strains such as TP117 and TR30 show significant growth suppression at 125 g/L corncob, Y117 maintains robust metabolic activity, underscoring its unique physiological adaptations to challenging fermentation conditions.

To contextualize Y117’s performance within the fungal CBP landscape, we compared its key metrics with representative engineered fungal platforms (Table 1). The Xyr1-overexpressing *Trichoderma reesei* QE2X strain, developed by Shen et al. (2022), achieved 37.87% utilization of acid-pretreated corncob residue at 50 g/L substrate loading [36]. In contrast, Y117 attained 72.2% utilization of untreated corncob at 125 g/L—representing not only higher efficiency but also operation at 2.5-fold higher substrate concentration without chemical pretreatment.

Similarly, recent metabolic engineering efforts in *Myceliophthora thermophila* have demonstrated CBP capability for organic acid production. Zhang et al. (2025) reported production of 4.5 g/L L-malic acid from 50 g/L microcrystalline cellulose following redox pathway engineering [17]. While this represents significant progress, the substrate loading (50 g/L) and product titer remain below Y117’s performance metrics. Furthermore, unlike thermophilic systems requiring elevated temperatures (45–50 °C), Y117 operates efficiently at 30 °C, offering potential energy savings.

The distinguishing features of Y117 that merit emphasis are: (i) exceptional high-solids degradation capability (72.2% at 125 g/L), significantly exceeding reported fungal CBP systems; (ii) inherent acid tolerance (maintaining enzymatic activity below pH 4.0), a critical trait for organic acid production that is lacking in many industrial strains; and (iii) rapid enzyme secretion kinetics (peak activities by day 6), enabling shorter fermentation cycles. The combination of these traits in a single fungal chassis is uncommon and positions Y117 as a promising platform for industrial lignocellulosic biorefining.

### 3.4. Industrial Potential of Engineered Y117 for Lactic Acid Fermentation

**Figure 7:**
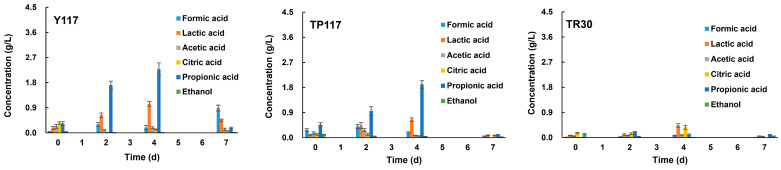
Time course of metabolite production by strains Y117, TP117, and TR30 in the cultures of corncob (125 g/L). Data are presented as mean ± SD (*n* = 3).

**Figure 8:**
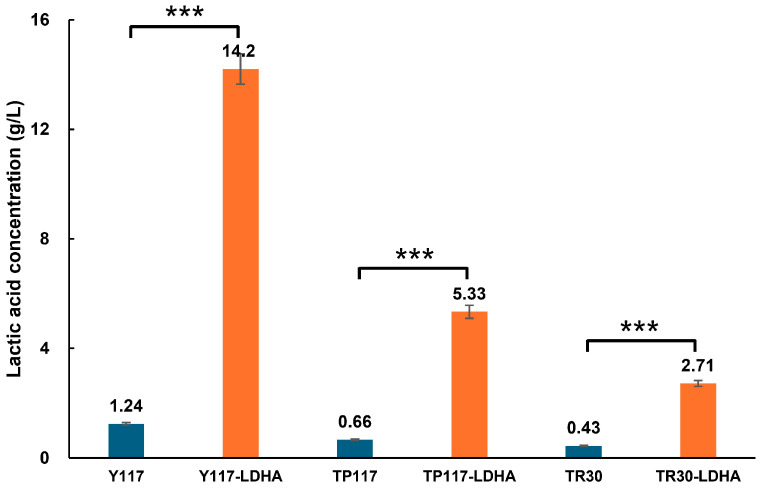
Lactic acid production before and after strain modification (125 g/L corncob). Statistical comparisons of lactic acid production between the parental and LDHA-engineered strains were performed using Welch’s two-tailed independent samples *t*-test. Data are presented as mean ± SD (*n* = 3). Asterisks indicate statistically significant differences (*** *p* < 0.001).

Using 125 g/L corncob powder as the sole carbon source, we initially assessed the metabolite production profiles of strains Y117, TP117, and TR30 at pH 6.0 (0, 2, 4, and 7 days) to characterize native fermentation patterns (Figure 7). Subsequently, LA fermentation experiments were conducted at pH 4.0 following *ldhA* expression, as this pH condition supports optimal cellulase stability and reduces contamination risk (Figure 8).

As shown in Figure 7, propionic acid emerged as the dominant metabolite in both Y117 and TP117, exhibiting characteristic biphasic kinetics: concentrations increased steadily during early fermentation, peaking at day 4 (Y117: 2.28 g/L; TP117: 1.91 g/L), followed by a decline to approximately 0.1 g/L by day 7. LA accumulated rapidly in Y117 and TP117 during the early fermentation phase, reaching 1.24 g/L and 0.66 g/L, respectively, by day 4, coinciding with the peak cellulase activity period (CMCase activity: 64.7 IU/mL for Y117 at day 4). In contrast, strain TR30 exhibited a distinct metabolite profile when cultivated on corncob, with LA emerging as the dominant product (0.43 g/L by day 4), which was lower than the peak concentration observed in Y117. Additionally, TR30 produced significant amounts of citric acid, accumulating to 0.37 g/L by day 4, a significantly higher level than those produced by Y117 and TP117. All three strains generated minor quantities of volatile fatty acids and solvents, including formic acid (0.07–0.35 g/L), acetic acid (0.09–0.50 g/L), and ethanol (0–0.10 g/L) throughout the fermentation period.

In order to enhance LA production, we introduced a single-copy plasmid harboring the lactate dehydrogenase A (*ldhA*) gene under the CBH1 promoter and CBH1 terminator (Figures S1–S5, Tables S1–S3). Western blot analysis confirmed successful expression of the LDHA protein in the engineered Y117 strain. A specific band at approximately 37 kDa, corresponding to the expected molecular weight of LDHA, was detected in Y117-LDHA extracts, whereas no signal was observed in the parental Y117 strain (Figure S6). This result demonstrates that the *ldhA* gene was successfully transcribed and translated into functional protein.

After the modification, all three strains cultivated on 125 g/L corncob substrate (initial pH 4.0, temperature 30 °C) showed significantly enhanced LA production (Figure 8). Y117 exhibited the most dramatic improvement, achieving 14.20 ± 0.55 g/L LA at 96 h, an 11.5-fold increase over the untransformed plasmid type (1.24 ± 0.04 g/L). The volumetric productivity for Y117 reached 0.15 g/L/h, with a carbon conversion efficiency improving from 1.8% to 20.4%. Engineered TP117 produced 5.33 ± 0.24 g/L (8-fold increase from 0.66 ± 0.02 g/L), whereas TR30 showed a more modest 6-fold improvement (from 0.43 ± 0.02 g/L to 2.71 ± 0.11 g/L) with 0.028 g/L/h productivity. The yield (lactic acid-g/corncob consumed-g) for Y117, TP117, and TR30 was 0.11, 0.04, and 0.02 g/g, respectively, highlighting Y117’s superior metabolic capability for consolidated bioprocessing applications.

In this study, the parental strain TP117 and the industrial benchmark TR30, both transformed with the same *ldhA* expression plasmid, served as comparative controls. This experimental design enables attribution of LA production enhancement to the combination of the genetic background (Y117 vs. TP117/TR30) and the *ldhA* cassette.

The expression plasmid was integrated into the *T. pinophilus* genome via single-crossover homologous recombination at the cbh1 promoter region (Section 2.7). This strategy inserts the *ldhA* expression cassette without disrupting the native cbh1 coding sequence, thereby preserving the integrity of the endogenous cellobiohydrolase I. Consequently, the cellulolytic complex remained intact, and the saccharification capacity of Y117-LDHA was not compromised by the genetic modification. This preserved cellulolytic function ensured that the superior substrate utilization of the parental Y117 was maintained after *ldhA* integration, supporting the substantial enhancement in LA production.

All three strains (Y117, TP117, and TR30) possess native LA biosynthesis pathways, as confirmed by their baseline production levels (1.24 g/L, 0.66 g/L, and 0.43 g/L, respectively) prior to the integration of the *ldhA* gene—a finding consistent with previous reports [23,40]. Following the introduction of a single-copy plasmid-based *ldhA* gene, LA titers increased significantly in all strains, though the extent of enhancement varied considerably: Y117 showed an 11.5-fold increase, TP117 an 8-fold, and TR30 a 6-fold increase. This divergence in performance reflects fundamental differences in substrate utilization efficiency among the strains. Y117 exhibited exceptional corncob degradation, achieving 72.2% utilization at 125 g/L solid loading, which supported high carbon flux toward lactate formation. In contrast, TR30 exhibited limited hydrolysis of cellulose and hemicellulose, as evidenced by significantly lower enzymatic activities (CMCase, FPase, and xylanase), along with notably low cellobiase activity as previously documented [41]. These constraints in enzymatic machinery reduced the availability of fermentable sugars, particularly under high-solids conditions, thereby restricting the potential for LA production.

The limited improvement in lactate production observed in TR30 (3-fold increase, reaching 2.71 g/L) can be attributed not only to its inherently poor substrate utilization but also to its well-documented sensitivity to acidic environments [42,43]. As LA accumulated and the pH dropped below 4.0, the β-glucosidase activity of TR30 was severely inhibited—a sharp contrast to the acid-tolerant *T. pinophilus*, which maintained robust enzyme performance under similar conditions, as documented in previous studies [22,44]. This pH-induced suppression of key enzymatic function explains the stagnant productivity of TR30 (0.028 g/L/h), even after *ldhA* overexpression, highlighting the critical role of acid tolerance in the effectiveness of engineered industrial strains for LA production.

Although Y117’s engineered lactate titer (14.20 g/L) and productivity (0.15 g/L/h) remain below commercial bacterial benchmarks (e.g., *Lactobacillus* spp., which can exceed 80 g/L), and direct comparison with such homofermentative bacteria is limited by inherent biological differences [45], its consolidated bioprocessing capability—simultaneously degrading lignocellulose and producing lactate without the need for exogenous enzymes—offers a promising one-step fermentation route with a distinct value proposition. The relatively low yield (Y = 0.11 g/g) is likely attributable to flux competition from alternative pathways, such as propionate synthesis. These results suggest that carbon was only partially directed toward lactate formation, which is consistent with the distributed carbon metabolism typically observed in filamentous fungi [46,47]. Nevertheless, Y117’s high plasmid stability underscores its strong potential as a chassis for further metabolic engineering. Ongoing strategies, including the knockout of propionate-related genes, are expected to substantially elevate lactate titers, with the goal of surpassing 50 g/L thus bringing the strain closer to industrial feasibility. A major technical bottleneck, however, remains the lack of robust genome-editing tools for Y117. Widely applied systems, such as CRISPR-Cas9 and Cre-loxP, have yet to be established in this strain, which complicates precise and efficient genetic manipulation. Overcoming this limitation is a key focus of our ongoing research and will be crucial to unlocking the full metabolic potential of Y117.

## 4. Conclusions

This study demonstrates that combining UV–NTG mutagenesis with plasmid-based metabolic engineering provides an effective strategy to construct high-performance fungal strains for lignocellulosic bioconversion. In line with our objectives, the engineered *T. pinophilus* Y117 exhibited strong cellulase secretion (FPase 8.9 IU/mL in 6 days; CMCase 64.7 IU/mL, 21.5-fold higher than the parental strain), robust growth, and superior corncob utilization rate (>60% across 50–125 g/L, peaking at 72.2% at 125 g/L) under high-solids conditions, outperforming both TP117 and the industrial benchmark *T. reesei* Rut-C30. Heterologous expression of the *ldhA* gene enabled Y117 to convert lignocellulosic sugars into LA, achieving 14.20 g/L LA with a productivity of 0.15 g/L/h. While LA productivity is below that of conventional bacterial strains, Y117’s consolidated bioprocessing capability eliminates the need for expensive enzyme supplementation and pretreatment, offering a cost-effective solution for next-generation industrial LA production. These results confirm that Y117 is a promising CBP chassis for lignocellulosic LA production, and further metabolic engineering, particularly targeting byproduct pathways and stress tolerance, will be crucial to maximizing its industrial potential.

## Figures and Tables

**Table 1:** Fungal CBP System Comparison.

Strain	Substrate	Concentration (g/L)	Utilization (%)	Product	Titer (g/L)	Productivity (g/L/h)	pH Tolerance	Ref
*T. pinophilus* Y117 (this study)	Corncob	125	72.2	LA	14.2	0.15	<4.0	-
*T. reesei* QE2X	Corncob residue	50	37.9	Enzymes	N/A	N/A	>5.0	[36]
*M. thermophila* engineered	Avicel	50	~40	Malic acid	4.5	0.03	~5.0	[17]
*A. brasiliensis ldhA*+	Glucose/xylose	20	N/A	LA	1.6	0.011	~6.0	[11]
*T. reesei* Rut-C30	Corncob	125	43.1	LA	2.7	0.028	~5.0	This study

## Data Availability

The original contributions presented in this study are included in the article/Supplementary Material. Further inquiries can be directed to the corresponding author.

## Supplementary Materials

The following supporting information can be downloaded at: https://www.mdpi.com/article/10.3390/jof12060405/s1, Figure S1: The plasmid map of pBIP-TPcbh1-LDHA-TPpk-hyg. *Hind*III and *Bam*HI were used to linearize the expression cassette for transforming *T. pinophilus* protoplasts; Figure S2: The plasmid map of pBIP-TR30cbh1-LDHA-TR30pk-hyg. *Sa*lI and *Bam*HI were used to linearize the expression cassette for transforming *T. reesei* protoplasts. Figure S3: (A) Protoplasts of *T. pinophilus* prepared for transformation with plasmid pBIP-TPcbh1-LDHA-TPpk-hyg. (B) Positive colonies obtained after transformation and selection on YPSA plates containing 500 μg/mL hygromycin. Figure S4: Diagnostic PCR results of *ldhA* gene using primer pair ldha-F/ldha-R in strains of Y117, TP117 and TR30; Figure S5: The colonies on plate medium supplemented with corncob (20 g/L) and bromocresol green (an indicator turns yellow in an acidic environment) before and after heterologous expression of the *ldhA* gene in Y117, TP117, and TR30; Figure S6. Western blot for LDHA-6×His-tag. Identification of the expression of *Rhizopus oryzae* derived *ldhA* gene in Y117-LDHA strain by Western blot using anti-His tag monoclonal antibody. Table S1: Primers used in this study; Table S2: Sequences of *ldhA* expression cassette for *T. pinophilus*. Table S3: Sequences of *ldhA* expression cassette for *T. reesei*.

## Abbreviations

The following abbreviations are used in this manuscript:

CBP	Consolidated bioprocessing
NTG	N-methyl-N′-nitro-N-nitrosoguanidine
LA	lactic acid
PDA	Potato Dextrose Agar
FPase activity	Filter-paper activity
CMCase activity	Carboxymethylcellulase activity
DNS	dinitrosalicylic acid
MTT	3-(4,5-dimethylthiazol-2-yl)-2,5-diphenyltetrazolium bromide
