# Supplementary material for: Synergistic Enhancement of Straw Hydrolysis and Lactic Acid Production in Talaromyces pinophilus Through Combined Random Mutagenesis and Plasmid Reconstruction
Source: J Fungi (Basel). 2026 Jun 3;12(6):405. doi: 10.3390/jof12060405 (PMC13301157; doi:10.3390/jof12060405)
Supplement: Supplementary file 1 [file jof-12-00405-s001.zip › jof-4280729-supplementary.pdf]

Supplementary Materials

Synergistic Enhancement of Straw Hydrolysis and Lactic Acid Production in *Talaromyces pinophilus* through Combined Random Mutagenesis and Plasmid Reconstruction

Siyuan Yue †, Ya Li †, Peng Li, Jing Zeng, Junhui Nie, Cheng Zhang, Tong Wang, Jianjun Guo, and Lin Yuan *

Institute of Biomanufacturing, Jiangxi Academy of Sciences, Nanchang 330095, China;

***Corresponding Author:** Lin Yuan
Institute of Biomanufacturing, Jiangxi Academy of Sciences, Nanchang 330095, China
E-mail: Yuanlin2003cn@aliyun.com; Tel.: +86-791-88176232

†These authors contributed equally to this work.

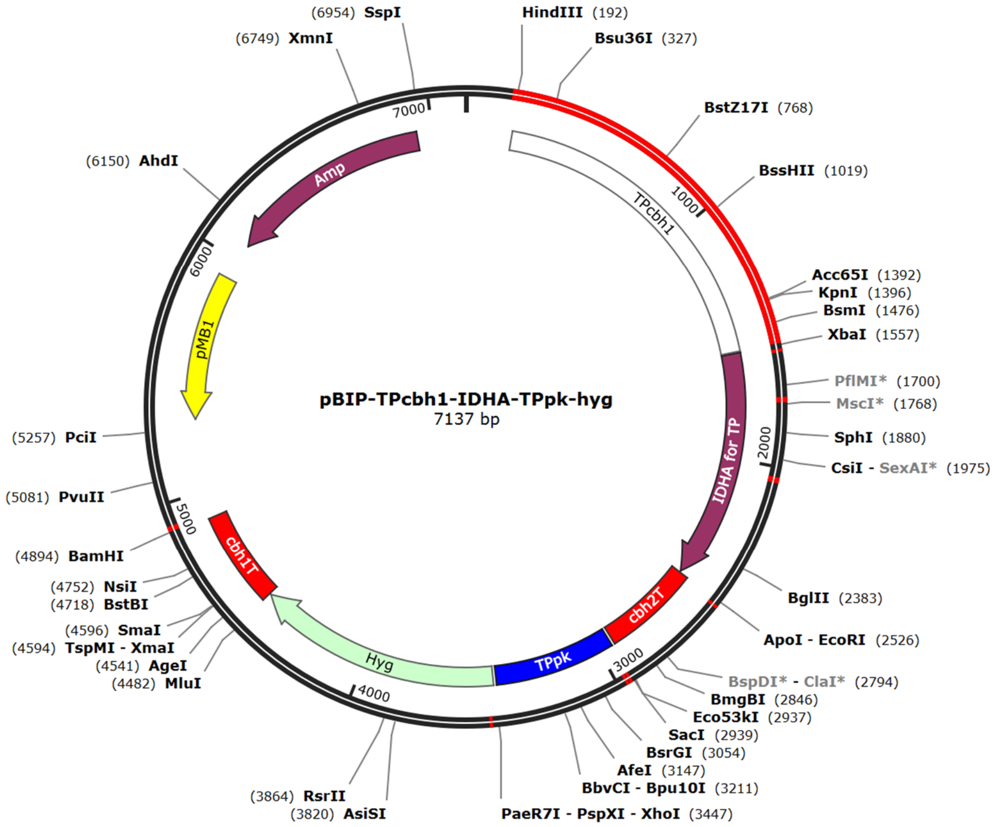

Figure S1. The plasmid map of pBIP-TPcbh1-LDHA-TPpk-hyg. *Hind*III and *Bam*HI were used to linearize the expression cassette for transforming *T. pinophilus* protoplasts.

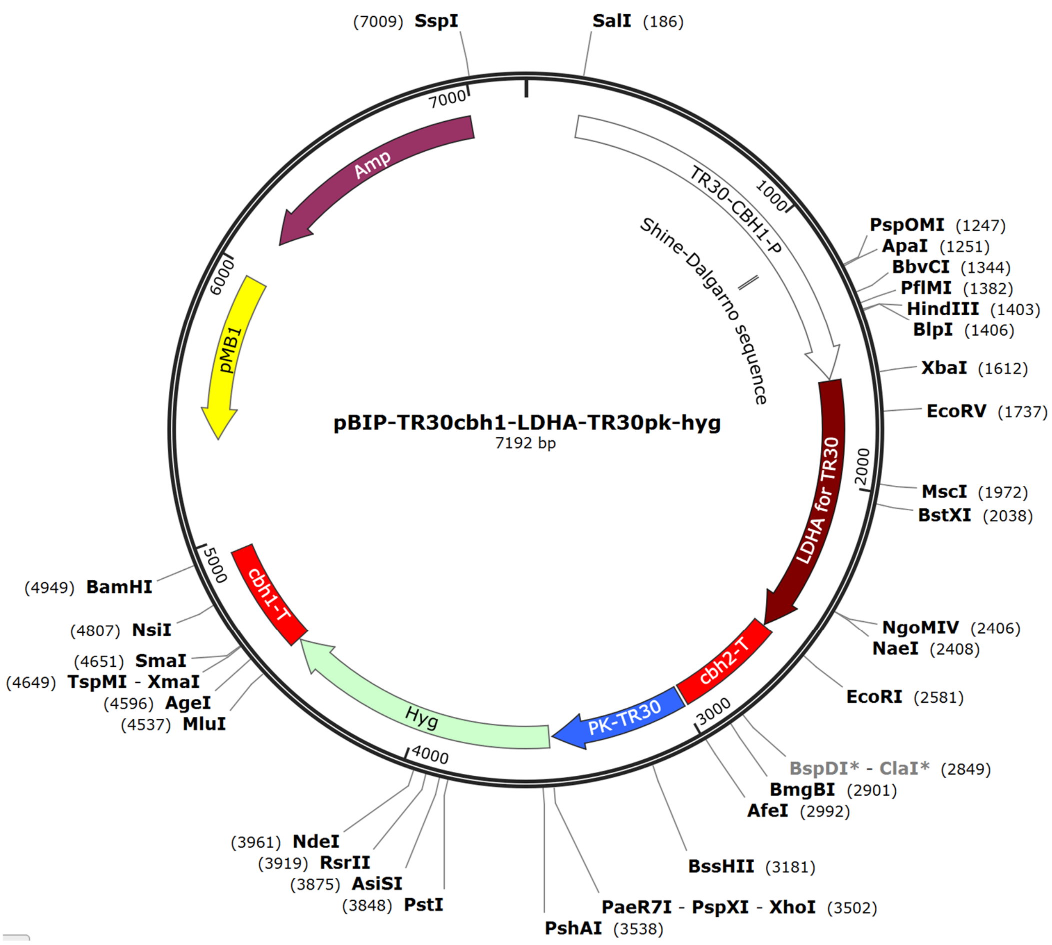

Figure S2. The plasmid map of pBIP-TR30cbh1-LDHA-TR30pk-hyg. *Sal*I and *Bam*HI were used to linearize the expression cassette for transforming *T. reesei* protoplasts.

The expression element TPcbh1 recombination arm and TP30cbh1 recombination arm are derived from the promoters of their respective exoglucanase I genes; the expression elements TPpk promoter and TR30pk promoter originate from the promoters of their respective pyruvate kinase genes; the expression elements cbh1T terminator and cbh2T terminator are derived from the terminators of the exoglucanase I/II genes of *T. reesei*.

Table S1. Primers used in this study.

**Name**	**Sequence (5’-3’)**	**Product/Purpose**
TPcbh1-F	actgagagtgcaccatgtcgacAAGCTTGGAA	TPcbh1 promoter /

	GCTCGTGAAAGC	recombination arm
TPcbh1-R	tctagaTGTGTCGATTGCTTCTGACTGTT	
TPldha-F	AAGCAATCGACACATCTAGAATGGTCCTGCACTCCAAGGT	LDHA gene for *T. pinophilus*
TPldha-R	GAATTCTTAGCAGGAGGACTTGGAGAAC	
Cbh2T-F1	AGTCCTCCTGCTAAgaattcggctttcgtgaccgggcttc	Cbh2 Terminator
Cbh2T-R1	agaGAGCTCtctatggaccagtacagccatgttgc	
TPpk-F	tgtactggtccatagaGAGCTCtctGTCAAGAATCGAATCTCATTGAAG	TPpk promoter
TPpk-R	gggCTCGAGcccTGTGAATGAGGGATCTCGTTCGG	
Hyg-F1	CATTCACAgggCTCGAGcccatgaagaagcctgaactcaccg	Hyg gene
Hyg-R1	ACGCGTctattcctttgccctcggacg	
Cbh1T-F1	gggcaaaggaatagACGCGTTGAACCCTTACTACTCTCAGTGCCT	Cbh1 Terminator
Cbh1T-R1	ccatgattacgccaagcggatccTGCTGCACATTGAACGATCATGAAATACCTTTGCC	
TR30cbh1-F	actgagagtgcaccatgtcgacACCTGTAAAGCCGCAATGCAGC	TR30cbh1 promoter / recombination arm
TR30cbh1-R	tctagaGATGCCAGTCCGCGGTTGACT	
TR30ldha-F	CGCGGACTGGCATCtctagaATGGTGCTGCACTCCAAAGTC	LDHA gene for TR30
TR30ldha-R	gaattcTTAACACGAACTTTTAGAAAAAGAGG	
Cbh2T-F2	AAAGTTCGTGTTAAgaattcggctttcgtgaccgggcttcaa	Cbh2 Terminator
Cbh2T-R2	agaagcgcttctatggaccagtacagccatgttg	
TR30pk-F	tggtccatagaagcgcttctGAGCGTTGCTGTGAGACCA	TR30pk promoter
TR30pk-R	gggCTCGAGcccGGTTAAGAGGGTTCTTCCGGCT	
Hyg-F2	TTAACCgggCTCGAGcccatgaagaagcctgaactcaccg	Hyg gene
Hyg-R2	ACGCGTctattcctttgccctcggacga	
Cbh1T-F2	gggcaaaggaatagACGCGTTGAACCCTTACTACTCTCAGTGCCTGTAAAG	Cbh1 Terminator
Cbh1T-R2	ccatgattacgccaagcggatccTGCTGCACATTGAACGATCATGAAATACCTTTG	

ldha-F	GGCTCCGCTGAAGAGGCTGG	Universal primers for the detection of the *ldhA* gene, producing a 309 bp amplicon
ldha-R	CTGGGGGTTGACGTCGAAGAC	

Table S2. Sequences of *ldhA* expression cassette for *T. pinophilus*.

pBIP-TPcbh1-LDHA-TPpk-hyg
tcgcgcgtttcggtgatgacggtgaaaacctctgacacatgcagctcccggagacggtcacagcttgtctgtaagcggatg<br>ccgggagcagacaagcccgtcagggcgcgtcagcgggtgttggcgggtgtcggggctggcttaactatgcggcatcag<br>agcagattgtactgagagtgcaccatgtcgacAAGCTTGGAAGCTCGTGAAAGCTGCCCTCA<br>CAATGATCGTCAAGATGACGTAGTTTGACTGGGTCGTTCCTGGATAAGGGT<br>TAGGGTAAATAGGGCTCAAAGTACCACGTGAGTGTGGAAAGATAAGCCCTA<br>ACCCTAAGGTCGTGTCGGACAAAAATTATCACTTGACCAAAATTGGAGATC<br>CCCTTAATGGAGCTTTTTGGTAATGGTTTGTATAGGGTTATGTGACGTCCGTA<br>TCACATGATTTTTATCCCAACAGGTCGATCCCCCTCTTATAGTTAATGGACA<br>ACATATAAGTACGTAGCATCTTAGATAGTTCGTCAGCGTCAACTGACCAAAG<br>TCCCCGTGTTTCATTTTAATTTGTCAGACTGCAAGAGTCTCGAAACATAAAA<br>AGATCGAAAGTTTTGCCTTATTAGGCTATGAGCATGAATGTCGGAACAATGC<br>CGTTGAGGCTATTCCCAATTTCGGAAATATGTATCTTCATTGCTGTCGACTTG<br>ACGACAGTCGATAAAAGGCTCATCCGGATAGATAAGCCAGATCACTCATTAT<br>GCCAATTTCTCCGGTGTCTGAAAACGTATACTACATACGTAACTGATCTTCG<br>TGGTTGAAAGAGTGTTTCTTCTCATTCTCATCTGCCGATGCCGAGCCAATTG<br>GAACAAACCCCGCATATGGTCCTGAATATCAATCGCGGAGATGCGGAGAGT<br>GAGGGAGCAACACAATTTTAAATTAGTCAGTTTTCTCATGTTCTCCGCAATC<br>TTGCAGGCTTGGGTCTGGTAGGTTTATCTCTCTCTTTTCACAACAAGGTTGG<br>GCCATTGTCAGCTTAGCAAGCGCGCAGCAAAGGGTGTCGGTCAATGTTCAT<br>GTCCTCCGCGGTCACTACAAAACAGCACGTGGGGAATGTTGCTTTCCCTGT<br>TGATGTTCATGTGTTGTCATTCCCGGCAAATCGACTCCAATTAATATGGTAG<br>GCTCCTGCATAATGCAAGTCCTTGAGATGCAGCTTCCGGCAGATGGACGTAT<br>AGATCAGGGACTTTGAGGGGCTAAAACACTTACCCGAGCTAAAACATACCA<br>TAATTTCGTTTAATGACTTTCGTCTGGATGGCAGAGGCTGAAGGTCGATTAT<br>GAGTGAAATTGGTATGAAGCCACATACGCCGATACTGTAACGCTGTGCCTT<br>CATCCGCCTTCTATCGCGCCCCGACATTCCGCGGTACCGCGATTATGAAGAA<br>AAACACTCGTAATGATGAGGAGATAGTTCTTAGCTCTTATTATTTTTGTCTAG<br>CTATGGAATGCAAGTTTAGCACTATATAATGGTGGTGTTTCCCTTGAAGAAT<br>TAGGCACTATCAACCGCAACAGTCAGAAGCAATCGACACAtctagaATGGTCC<br>TGCACTCCAAGGTCGCCATCGTCGGCGCCGGCGCTGTCGGTGCTTCCACTG<br>CTTACGCCCTGATGTTCAAGAACATCTGCACCGAGATCATCATCGTCGATGT<br>CAACCCGGACATCGTCCAGGCCCAGGTCCTGGACCTGGCCGACGCTGCTT<br>CGATCTCCCACACCCCCATCCGCGCCGGCTCCGCTGAAGAGGCTGGCCAG<br>GCTGACATCGTCGTCATCACCGCCGGCGCCAAGCAGCGCGAGGGTGAACC<br>TCGCACCAAGCTGATCGAGCGCAACTTCCGCGTCCTGCAGTCGATCATCGG

CGGCATGCAGCCGATCCGCCCCGACGCTGTCATCCTGGTCGTCGCCAACCC
GGTCGATATCCTGACCCACATCGCCAAGACCCTGTCCGGCCTGCCCCCGAA
CCAGGTCATCGGCTCGGGCACCTACCTGGACACCACCCGCCTGCGCGTCCA
CCTGGGTGACGTCTTCGACGTCAACCCCCAGTCCGTCCACGCCTTCGTCCT
GGGCGAGCACGGCGACTCGCAGATGATCGCCTGGGAGGCCGCCTCCATCG
GCGGTCAGCCTCTGACCTCCTTCCCCGAGTTCGCCAAGCTGGACAAGACC
GCCATCTCCAAGGCCATCTCGGGCAAGGCCATGGAGATCATCCGCCTGAAG
GGCGCCACCTTCTACGGCATCGGCGCCTGCGCCGCCGACTTGGTCCACACT
ATCATGCTGAACCGCAAGTCGGTCCACCCGGTCTCGGTCTACGTCGAGAAG
TACGGCGCCACCTTTTCCATGCCGGCCAAGCTGGGCTGGCGCGGTGTCGAA
CAGATCTACGAGGTCCCCCTGACCGAGGAGGAGGCCCTGCTGGTCAA
GTCCGTCGAGGCCCTGAAGTCCGTCGAATACTCGTCCACTAAGGTCCCCGA
GAAGAAGGTCCACGCCACCTCGTTCTCCAAGTCCTCCTGCTAAgaattcggctttc
gtgaccgggcttcaaacaatgatgtgcgatggtgtgattcccggttggcggagtctttgtctactttggttgtctgtcgcaggt
cggtagaccgcaaatgagcaactgatggattgttgccagcgatactataattcacatggatggtctttgtcgatcagtagcta
gtgagagagagagaacatctatccacaatgtcgagtgtctattagacatattccgagaataaagtcaaccgtgtctgtgatct
aaagatcgattcggcagtcgagtagcgtataacaactccgagtaaccagcaaaagcacgtcgtgacaggagcagggcttt
gccaactgcgcaaccttgcttgaatgaggatacacgggttgcaacatggctgtactggtccatagaGAGCTCtctGT
CAAGAATCGAATCTCATTGAAGAGAGCCAGAGACTCAAGAGTGCAATTGT
GATAGATAAGATAAATCAATGTTTCTGGGAGAGTATTGCAGTATATACTGTAT
ATAACTGTACAAGTATATAATAATAATTGATAATAAATGCAGAAGATAGAAG
GGCAGAAAACCGCCGGAGATATCCAGGAAAAAAAAGAAAAGTTCAGCGC
TAAGCCTAATTGAGCGCCGGACCACTGGCATTTTTGGGAGCGCCCACGGTG
GTTCTTTCCCCTCAGCAGCTTTTCCTGACCCTGCGACAAATCTTATTTTCTC
CTGCTATCTCCCTTGCTTCTCCCTCTCTCTTCTCGTCTTCCATCTCTCCCATA
ACTCTTACACTTACATTCCTCACTCCTGCATACATTTCCATTTGGATAGATCC
TCTCTGTCCAGGTATTGCAGCAGTCCTTAGTTTCCGGCACCTCTTTTTGCTC
CTCTTGTTCTCCGAACGAGATCCCTCATTCACAgggCTCGAGcccatgaagaagcctg
aactcaccgcgacgtctgtcgagaagtttctgatcgaaaagttcgacagcgtctccgacctgatgcagctctcggagggcg
aagaatctcgtgctttcagcttcgatgtaggagggcgtggatatgtcctgcgggtaaatagctgcgccgatggtttctacaaa
gatcgttatgtttatcggcactttgcatcggccgcgctcccgattccggaagtgcttgacattggggagttcagcgagagcct
gacctattgcatctcccgccgtgcacagggtgtcacgttgcaagacctgcctgaaaccgaactgcccgctgttctgcagcc
ggtcgcggaggccatggatgcgatcgctgcggccgatcttagccagacgagcgggttcggcccattcggaccgcaagg
aatcggtcaatacactacatggcgtgatttcatatgcgcgattgctgatccccatgtgtatcactggcaaactgtgatggacga
caccgtcagtgcgtccgtcgcgcaggctctcgatgagctgatgctttgggccgaggactgccccgaagtccggcacctcg
tgcacgcggatttcggctccaacaatgtcctgacggacaatggccgcataacagcggtcattgactggagcgaggcgatg
ttcggggattcccaatacgaggtcgccaacatcttcttctggaggccgtggttggcttgtatggagcagcagacgcgctactt
cgagcggaggcatccggagcttgcaggatcgccgcggctccgggcgtatatgctccgcattggtcttgaccaactctatca
gagcttggttgacggcaatttcgatgatgcagcttgggcgcagggtcgatgcgacgcaatcgtccgatccggagccggga
ctgtcgggcgtacacaaatcgcccgcagaagcgcggccgtctggaccgatggctgtgtagaagtactcgccgatagtgg
aaaccgacgccccagcactcgtccgagggcaaaggaatagACGCGTTGAACCCTTACTACTCTCA
GTGCCTGTAAAGCTCCGTGGCGAAAGCCTGACGCACCGGTAGATTCTTGGT
GAGCCCGTATCATGACGGCGGCGGGAGCTACATGGCCCCGGGTGATTTATT
TTTTTTGTATCTACTTCTGACCCTTTTCAAATATACGGTCAACTCATCTTTCA

CTGGAGATGCGGCCTGCTTGGTATTGCGATGTTGTCAGCTTGGCAAATTGT
GGCTTTCGAAAACACAAAACGATTCCTTAGTAGCCATGCATTTTAAGATAA
CGGAATAGAAGAAAGAGGAAATTAAAAAAAAAAAAAAAAACAAACATCCC
GTTCATAACCCGTAGAATCGCCGCTCTTCGTGTATCCCAGTACCACGGCAA
AGGTATTTCATGATCGTTCAATGTGCAGCAggatccgcttggcgtaatcatggtcatagctgtttc
ctgtgtgaaattgttatccgctcacaattccacacaacatacgagccggaagcataaagtgtaaagcctggggtgcctaatg
agtgagctaactcacattaattgcgttgcgctcactgcccgctttccagtcgggaaacctgtcgtgccagctgcattaatgaat
cggccaacgcgcggggagaggcggtttgcgtattgggcgctcttccgcttcctcgctcactgactcgctgcgctcggtcgt
tcggctgcggcgagcggtatcagctcactcaaaggcggtaatacggttatccacagaatcaggggataacgcaggaaag
aacatgtgagcaaaaggccagcaaaaggccaggaaccgtaaaaaggccgcgttgctggcgtttttccataggctccgccc
ccctgacgagcatcacaaaaatcgacgctcaagtcagaggtggcgaaacccgacaggactataaagataccaggcgtttc
cccctggaagctccctcgtgcgctctcctgttccgaccctgccgcttaccggatacctgtccgcctttctcccttcgggaagc
gtggcgctttctcatagctcacgctgtaggtatctcagttcggtgtaggtcgttcgctccaagctgggctgtgtgcacgaacc
ccccgttcagcccgaccgctgcgccttatccggtaactatcgtcttgagtccaacccggtaagacacgacttatcgccactg
gcagcagccactggtaacaggattagcagagcgaggtatgtaggcggtgctacagagttcttgaagtggtggcctaacta
cggctacactagaagaacagtatttggtatctgcgctctgctgaagccagttaccttcggaaaaagagttggtagctcttgat
ccggcaaacaaaccaccgctggtagcggtggtttttttgtttgcaagcagcagattacgcgcagaaaaaaaggatctcaag
aagatcctttgatcttttctacggggtctgacgctcagtggaacgaaaactcacgttaagggattttggtcatgagattatcaaa
aaggatcttcacctagatccttttaaattaaaaatgaagttttaaatcaatctaaagtatatatgagtaaacttggtctgacagtta
ccaatgcttaatcagtgaggcacctatctcagcgatctgtctatttcgttcatccatagttgcctgactccccgtcgtgtagataa
ctacgatacgggagggcttaccatctggccccagtgctgcaatgataccgcgagacccacgctcaccggctccagatttat
cagcaataaaccagccagccggaagggccgagcgcagaagtggtcctgcaactttatccgcctccatccagtctattaatt
gttgccgggaagctagagtaagtagttcgccagttaatagtttgcgcaacgttgttgccattgctacaggcatcgtggtgtca
cgctcgtcgtttggtatggcttcattcagctccggttcccaacgatcaaggcgagttacatgatcccccatgttgtgcaaaaaa
gcggttagctccttcggtcctccgatcgttgtcagaagtaagttggccgcagtgttatcactcatggttatggcagcactgcat
aattctcttactgtcatgccatccgtaagatgcttttctgtgactggtgagtactcaaccaagtcattctgagaatagtgtatgcg
gcgaccgagttgctcttgcccggcgtcaatacgggataataccgcgccacatagcagaactttaaaagtgctcatcattgga
aaacgttcttcggggcgaaaactctcaaggatcttaccgctgttgagatccagttcgatgtaacccactcgtgcacccaactg
atcttcagcatcttttactttcaccagcgtttctgggtgagcaaaaacaggaaggcaaaatgccgcaaaaaagggaataagg
gcgacacggaaatgttgaatactcatactcttcctttttcaatattattgaagcatttatcagggttattgtctcatgagcggatac
atatttgaatgtatttagaaaaataaacaaataggggttccgcgcacatttccccgaaaagtgccacctgacgtctaagaaac
cattattatcatgacattaacctataaaaataggcgtatcacgaggccctttcgtc

Table S3. Sequences of *ldhA* expression cassette for *T. reesei*.

pBIP-TR30cbh1-LDHA-TR30pk-hyg
tcgcgcgtttcggtgatgacggtgaaaacctctgacacatgcagctcccggagacggtcacagcttgtctgtaagcggatg ccgggagcagacaagcccgtcagggcgcgtcagcgggtgttggcgggtgtcggggctggcttaactatgcggcatcag agcagattgtactgagagtgcaccatgtcgacACCTGTAAAGCCGCAATGCAGCATCACTGGA AAATACAAACCAATGGCTAAAAGTACATAAGTTAATGCCTAAAGGAGTCAT ATACCAGCGGCTAATAATTGTACAATCAAGTGGCTAAACGTACCGTAATTTG CCAACGGCTTCTTCACTCAGTCCAATCTCAGCTGGTGATCCCCCAATTGGG TCGCTTGTTTGTTCCACCTGTAAAGCCGCAATGCAGCATCACTGGAAAATA

CAAACCAATGGCTAAAAGTACATAAGTTAATGCCTAAAGGAGTCATATACC
AGCGGCTAATAATTGTACAATCAAGTGGCTAAACGTACCGTAATTTGCCAAC
GGCTTCTTCACTCAGTCCAATCTCAGCTGGTGATCCCCCAATTGGGTCGCTT
GTTTGTTCCACCTGTAAAGCCGCAATGCAGCATCACTGGAAAATACAAACC
AATGGCTAAAAGTACATAAGTTAATGCCTAAAGGAGTCATATACCAGCGGCT
AATAATTGTACAATCAAGTGGCTAAACGTACCGTAATTTGCCAACGGCTTCT
TCACTCAGTCCAATCTCAGCTGGTGATCCCCCAATTGGGTCGCTTGTTTGTT
CCACCTGTAAAGCCGCAATGCAGCATCACTGGAAAATACAAACCAATGGCT
AAAAGTACATAAGTTAATGCCTAAAGGAGTCATATACCAGCGGCTAATAATT
GTACAATCAAGTGGCTAAACGTACCGTAATTTGCCAACGGCTTCTTCACTC
AGTCCAATCTCAGCTGGTGATCCCCCAATTGGGTCGCTTGTTTGTTCCGGTG
AAGTGAAAGAAGACAGAGGTAAGAATGTCTGACTCGGAGCGTTTTGCATA
CAACCAAGGGCAGTGATGGAAGACAGTGAAATGTTGACATTCAAGGAGTA
TTTAGCCAGGGATGCTTGAGTGTATCGTGTAAGGAGGTTTGTCTGCCGATAC
GACGAATACTGTATAGTCACTTCTGATGAAGTGGTCCATATTGAAATGTAAG
TCGGCACTGAACAGGCAAAAGATTGAGTTGAAACTGCCTAAGATCTCGGG
CCCTCGGGCCTTCGGCCTTTGGGTGTACATGTTTGTGCTCCGGGCAAATGC
AAAGTGTGGTAGGATCGAACACACTGCTGCCTTTACCAAGCAGCTGAGGG
TATGTGATAGGCAAATGTTCAGGGGCCACTGCATGGTTTCGAATAGAAAGA
GAAGCTTAGCCAAGAACAATAGCCGATAAAGATAGCCTCATTAAACGGAAT
GAGCTAGTAGGCAAAGTCAGCGAATGTGTATATATAAAGGTTCGAGGTCCG
TGCCTCCCTCATGCTCTCCCCATCTACTCATCAACTCAGATCCTCCAGGAGA
CTTGTACACCATCTTTTGAGGCACAGAAACCCAATAGTCAACCGCGGACTG
GCATCtctagaATGGTGCTGCACTCCAAAGTCGCGATTGTTGGTGCTGGAGCC
GTTGGGGCCTCAACTGCCTACGCGCTAATGTTCAAGAACATCTGCACGGAG
ATCATTATTGTGGACGTCAATCCAGATATCGTGCAGGCCCAAGTGCTCGATC
TCGCAGACGCCGCGTCAATCTCGCATACCCCCATCCGTGCAGGCTCCGCTG
AAGAGGCTGGACAAGCCGACATCGTCGTCATAACGGCTGGAGCGAAGCAG
CGAGAGGGCGAACCGCGCACCAAGCTGATTGAGCGGAATTTCCGCGTCCT
TCAGAGCATCATCGGCGGCATGCAGCCTATACGGCCTGACGCTGTTATTCTT
GTCGTGGCCAACCCCGTTGACATCTTGACACACATCGCGAAGACCCTGTCG
GGCCTGCCCCCAAACCAAGTCATTGGGAGCGGGACTTACCTTGACACCAC
CCGATTGAGGGTGCATCTGGGCGATGTCTTCGACGTCAACCCCCAGAGCGT
CCACGCCTTCGTCCTGGGCGAGCACGGCGACAGCCAGATGATTGCCTGGG
AGGCGGCCTCGATAGGTGGACAGCCGCTCACTAGCTTCCCCGAGTTTGCAA
AGCTCGACAAGACGGCGATCTCCAAGGCGATTAGCGGCAAGGCCATGGAA
ATCATCCGCCTGAAGGGCGCTACGTTTTACGGCATTGGCGCATGCGCCGCC
GATCTGGTCCACACAATCATGCTCAACAGGAAGTCGGTGCATCCCGTGTCT
GTTTATGTCGAAAAGTACGGGGCAACGTTCTCCATGCCGGCTAAGTTGGGC
TGGAGAGGTGTTGAGCAGATCTACGAGGTGCCTCTCACCGAGGAGGAAGA
AGCCCTCCTCGTCAAGTCTGTAGAGGCCCTCAAGTCAGTCGAGTATTCCTC
CACAAAAGTACCGGAGAAGAAGGTCCACGCAACCTCTTTTTCTAAAAGTT
CGTGTTAAgaattcggctttcgtgaccgggcttcaaacaatgatgtgcgatggtgtgattcccggttggcggagtcttt
gtctactttggttgtctgtcgcaggtcggtagaccgcaaatgagcaactgatggattgttgccagcgatactataattcacatg

gatggtctttgtcgatcagtagctagtgagagagagagaacatctatccacaatgtcgagtgtctattagacatattccgaga
ataaagtcaaccgtgtctgtgatctaaagatcgattcggcagtcgagtagcgtataacaactccgagtaaccagcaaaagca
cgtcgtgacaggagcagggctttgccaactgcgcaaccttgcttgaatgaggatacacgggttgcaacatggctgtactgg
tccatagaagcgcttctGAGCGTTGCTGTGAGACCATGAGCTATTATTGCTAGGTACA
GTATAGAGAGAGGAGAGAGAGAGAGAGAGTGTGAGAGGGAAAAGGTGAG
GTTGAAGTGAGGTAGTAAGTGAAAAAAAAAAAAAAATCAACACTGACGG
CTGCCGTCTGCCACCCTCCTCCACCCAGCCACCTGCACACTCAGCGCGCAG
CATCACCTAATCTTGGCTCGCCTTCCGCAGCTCAGGTTGTTTTTTTTTTTCT
CTCTCCTCGTCGAAGCCGCCCTTGTTCCCTTATTTATTTCCCTCTCCTACCTT
GTCTGCCTTTGGTCCATCTGCCCCTTTGTCTGCATCTCTTTTGCACGCATCG
CCTTATCGTCGTCTCTTTTTTCACTCACGGGAGCTTGACGAAGACCTGACTC
GTGAGCCTCACCTGCTGATTTCTCTCCCCCCCTCCCGACCGGCTTGACTTTT
GTTTCTCCTCCAGTACCTTATCGCGAAGCCGGAAGAACCCTCTTAACCgggC
TCGAGcccatgaagaagcctgaactcaccgcgacgtctgtcgagaagtttctgatcgaaaagttcgacagcgtctccg
acctgatgcagctctcggagggcgaagaatctcgtgctttcagcttcgatgtaggagggcgtggatatgtcctgcgggtaa
atagctgcgccgatggtttctacaaagatcgttatgtttatcggcactttgcatcggccgcgctcccgattccggaagtgcttg
acattggggagttcagcgagagcctgacctattgcatctcccgccgtgcacagggtgtcacgttgcaagacctgcctgaaa
ccgaactgcccgctgttctgcagccggtcgcggaggccatggatgcgatcgctgcggccgatcttagccagacgagcgg
gttcggcccattcggaccgcaaggaatcggtcaatacactacatggcgtgatttcatatgcgcgattgctgatccccatgtgt
atcactggcaaactgtgatggacgacaccgtcagtgcgtccgtcgcgcaggctctcgatgagctgatgctttgggccgag
gactgccccgaagtccggcacctcgtgcacgcggatttcggctccaacaatgtcctgacggacaatggccgcataacagc
ggtcattgactggagcgaggcgatgttcggggattcccaatacgaggtcgccaacatcttcttctggaggccgtggttggct
tgtatggagcagcagacgcgctacttcgagcggaggcatccggagcttgcaggatcgccgcggctccgggcgtatatgc
tccgcattggtcttgaccaactctatcagagcttggttgacggcaatttcgatgatgcagcttgggcgcagggtcgatgcgac
gcaatcgtccgatccggagccgggactgtcgggcgtacacaaatcgcccgcagaagcgcggccgtctggaccgatggc
tgtgtagaagtactcgccgatagtggaaaccgacgccccagcactcgtccgagggcaaaggaatagACGCGTTG
AACCCTTACTACTCTCAGTGCCTGTAAAGCTCCGTGGCGAAAGCCTGACGC
ACCGGTAGATTCTTGGTGAGCCCGTATCATGACGGCGGCGGGAGCTACATG
GCCCCGGGTGATTTATTTTTTTTGTATCTACTTCTGACCCTTTTCAAATATAC
GGTCAACTCATCTTTCACTGGAGATGCGGCCTGCTTGGTATTGCGATGTTGT
CAGCTTGGCAAATTGTGGCTTTCGAAAACACAAAACGATTCCTTAGTAGCC
ATGCATTTTAAGATAACGGAATAGAAGAAAGAGGAAATTAAAAAAAAAAA
AAAAACAAACATCCCGTTCATAACCCGTAGAATCGCCGCTCTTCGTGTATCC
CAGTACCACGGCAAAGGTATTTCATGATCGTTCAATGTGCAGCAggatccgcttgg
cgtaatcatggtcatagctgtttcctgtgtgaaattgttatccgctcacaattccacacaacatacgagccggaagcataaagt
gtaaagcctggggtgcctaatgagtgagctaactcacattaattgcgttgcgctcactgcccgctttccagtcgggaaacctg
tcgtgccagctgcattaatgaatcggccaacgcgcggggagaggcggtttgcgtattgggcgctcttccgcttcctcgctca
ctgactcgctgcgctcggtcgttcggctgcggcgagcggtatcagctcactcaaaggcggtaatacggttatccacagaat
caggggataacgcaggaaagaacatgtgagcaaaaggccagcaaaaggccaggaaccgtaaaaaggccgcgttgctg
gcgtttttccataggctccgcccccctgacgagcatcacaaaaatcgacgctcaagtcagaggtggcgaaacccgacagg
actataaagataccaggcgtttccccctggaagctccctcgtgcgctctcctgttccgaccctgccgcttaccggatacctgt
ccgcctttctcccttcgggaagcgtggcgctttctcatagctcacgctgtaggtatctcagttcggtgtaggtcgttcgctcca
agctgggctgtgtgcacgaaccccccgttcagcccgaccgctgcgccttatccggtaactatcgtcttgagtccaacccggt
aagacacgacttatcgccactggcagcagccactggtaacaggattagcagagcgaggtatgtaggcggtgctacagagt

tcttgaagtggtggcctaactacggctacactagaagaacagtatttggtatctgcgctctgctgaagccagttaccttcgga
aaaagagttggtagctcttgatccggcaaacaaaccaccgctggtagcggtggtttttttgtttgcaagcagcagattacgcg
cagaaaaaaaggatctcaagaagatcctttgatcttttctacggggtctgacgctcagtggaacgaaaactcacgttaaggg
attttggtcatgagattatcaaaaaggatcttcacctagatccttttaaattaaaaatgaagttttaaatcaatctaaagtatatatg
agtaaacttggtctgacagttaccaatgcttaatcagtgaggcacctatctcagcgatctgtctatttcgttcatccatagttgcc
tgactccccgtcgtgtagataactacgatacgggagggcttaccatctggccccagtgctgcaatgataccgcgagaccca
cgctcaccggctccagatttatcagcaataaaccagccagccggaagggccgagcgcagaagtggtcctgcaactttatc
cgcctccatccagtctattaattgttgccgggaagctagagtaagtagttcgccagttaatagtttgcgcaacgttgttgccatt
gctacaggcatcgtggtgtcacgctcgtcgtttggtatggcttcattcagctccggttcccaacgatcaaggcgagttacatg
atcccccatgttgtgcaaaaaagcggttagctccttcggtcctccgatcgttgtcagaagtaagttggccgcagtgttatcact
catggttatggcagcactgcataattctcttactgtcatgccatccgtaagatgcttttctgtgactggtgagtactcaaccaagt
cattctgagaatagtgtatgcggcgaccgagttgctcttgcccggcgtcaatacgggataataccgcgccacatagcagaa
ctttaaaagtgctcatcattggaaaacgttcttcggggcgaaaactctcaaggatcttaccgctgttgagatccagttcgatgt
aacccactcgtgcacccaactgatcttcagcatcttttactttcaccagcgtttctgggtgagcaaaaacaggaaggcaaaat
gccgcaaaaaagggaataagggcgacacggaaatgttgaatactcatactcttcctttttcaatattattgaagcatttatcag
ggttattgtctcatgagcggatacatatttgaatgtatttagaaaaataaacaaataggggttccgcgcacatttccccgaaaa
gtgccacctgacgtctaagaaaccattattatcatgacattaacctataaaaataggcgtatcacgaggccctttcgtc

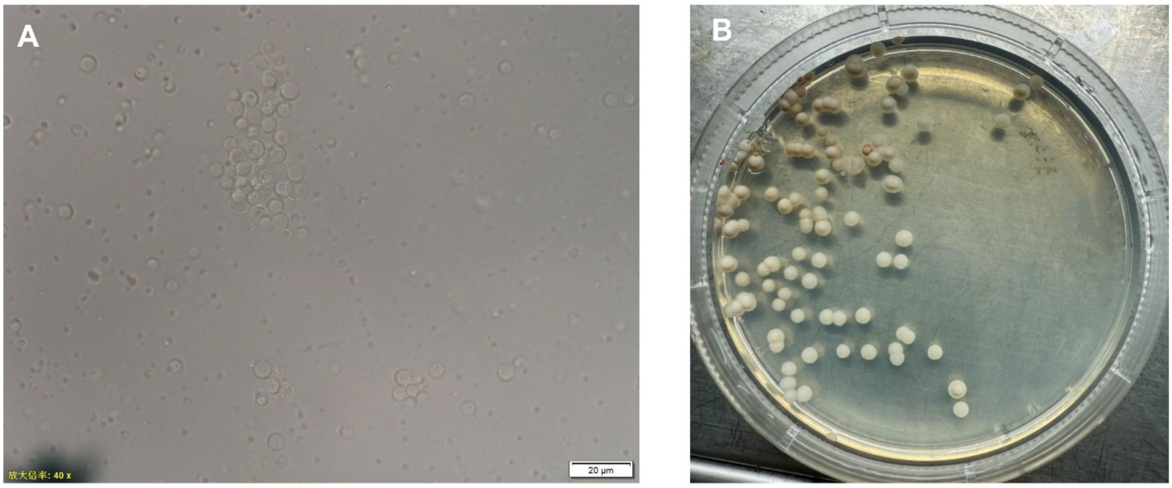

Figure S3. (A) Protoplasts of *T. pinophilus* prepared for transformation with plasmid pBIP-TPcbh1-LDHA-TPpk-hyg. (B) Positive colonies obtained after transformation and selection on YPSA plates containing 500 μg/mL hygromycin.

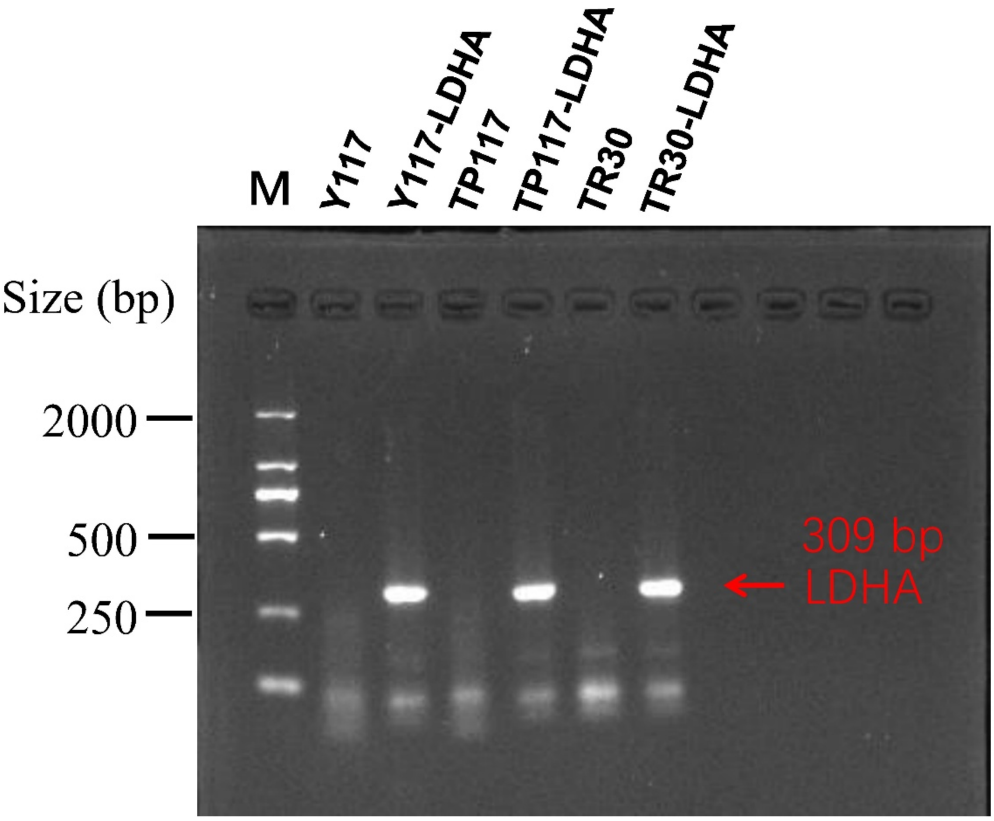

Figure S4. Diagnostic PCR results of *ldhA* gene using primer pair ldha-F/ldha-R in strains of Y117, TP117 and TR30.

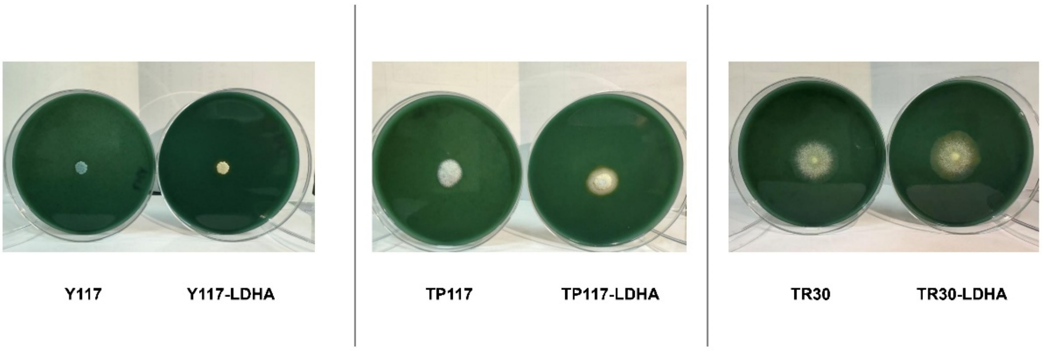

Figure S5. The colonies on plate medium supplemented with corncob (20 g/L) and bromocresol green (an indicator turns yellow in an acidic environment) before and after heterologous expression of the *ldhA* gene in Y117, TP117, and TR30.

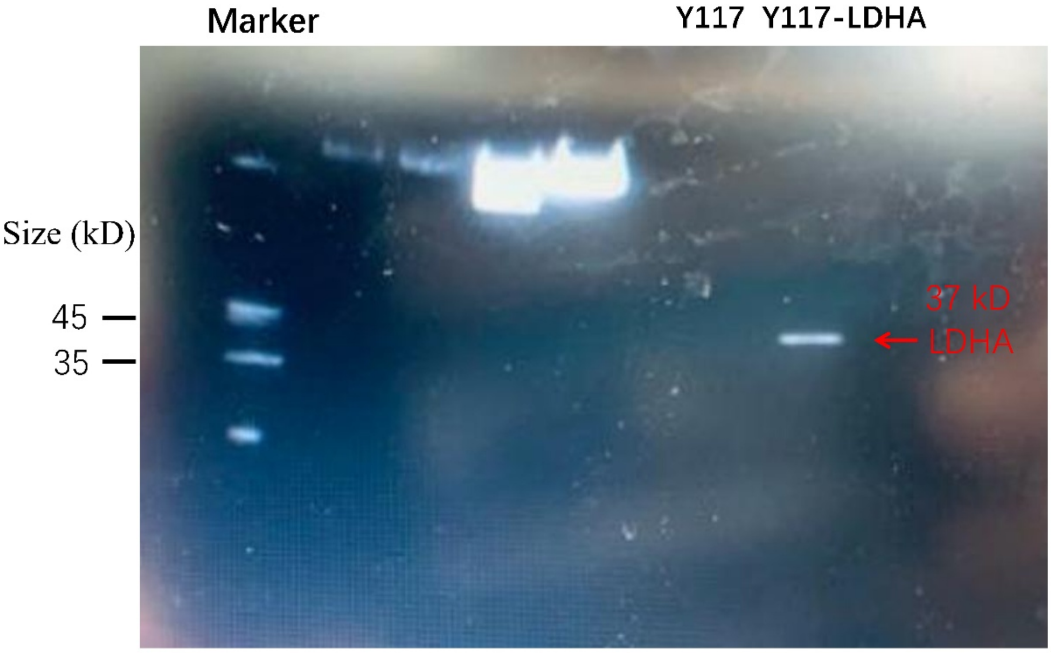

Figure S6. Western blot for LDHA-6×His-tag. Identification of the expression of *Rhizopus oryzae* derived *ldhA* gene in Y117-LDHA strain by Western blot using anti-His tag monoclonal antibody.
